# Supplementary material for: Pyruvate Dehydrogenase Kinase 1 inhibition mediated oxidative phosphorylation enhancement in cartilage promotes osteoarthritis progression
Source: BMC Musculoskelet Disord. 2023 Jul 20;24:597. doi: 10.1186/s12891-023-06585-6 (PMC10357736; doi:10.1186/s12891-023-06585-6)
Supplement: Supplementary file 1 — Additional file 1. [file 12891_2023_6585_MOESM1_ESM.zip › KEGG.Gsea.1653622432464/KEGG_GLYCOSAMINOGLYCAN_BIOSYNTHESIS_HEPARAN_SULFATE.html]

Details for gene set KEGG\_GLYCOSAMINOGLYCAN\_BIOSYNTHESIS\_HEPARAN\_SULFATE[GSEA]

|  || Dataset | 55457\_collapsed\_to\_symbols.cls.cls #OA\_versus\_normal.cls.cls #OA\_versus\_normal\_repos |
| Phenotype | cls.cls#OA\_versus\_normal\_repos |
| Upregulated in class | OA |
| GeneSet | KEGG\_GLYCOSAMINOGLYCAN\_BIOSYNTHESIS\_HEPARAN\_SULFATE |
| Enrichment Score (ES) | 0.3299903 |
| Normalized Enrichment Score (NES) | 1.0945321 |
| Nominal p-value | 0.33636364 |
| FDR q-value | 0.49020377 |
| FWER p-Value | 1.0 |
Table: GSEA Results Summary

  

Fig 1: Enrichment plot: KEGG\_GLYCOSAMINOGLYCAN\_BIOSYNTHESIS\_HEPARAN\_SULFATE      
 Profile of the Running ES Score & Positions of GeneSet Members on the Rank Ordered List

  

| SYMBOL | TITLE | RANK IN GENE LIST | RANK METRIC SCORE | RUNNING ES | CORE ENRICHMENT || 1 | EXT2 | exostosin glycosyltransferase 2 [Source:HGNC Symbol;Acc:HGNC:3513] | 170 | 0.838 | 0.1140 | Yes |
| 2 | EXTL2 | exostosin like glycosyltransferase 2 [Source:HGNC Symbol;Acc:HGNC:3516] | 357 | 0.722 | 0.2091 | Yes |
| 3 | B4GALT7 | "beta-1,4-galactosyltransferase 7 [Source:HGNC Symbol;Acc:HGNC:930]" | 1411 | 0.442 | 0.1944 | Yes |
| 4 | NDST2 | N-deacetylase and N-sulfotransferase 2 [Source:HGNC Symbol;Acc:HGNC:7681] | 1587 | 0.415 | 0.2438 | Yes |
| 5 | XYLT2 | xylosyltransferase 2 [Source:HGNC Symbol;Acc:HGNC:15517] | 1938 | 0.368 | 0.2725 | Yes |
| 6 | HS3ST3A1 | heparan sulfate-glucosamine 3-sulfotransferase 3A1 [Source:HGNC Symbol;Acc:HGNC:5196] | 2637 | 0.286 | 0.2618 | Yes |
| 7 | B3GAT3 | "beta-1,3-glucuronyltransferase 3 [Source:HGNC Symbol;Acc:HGNC:923]" | 2987 | 0.251 | 0.2728 | Yes |
| 8 | EXT1 | exostosin glycosyltransferase 1 [Source:HGNC Symbol;Acc:HGNC:3512] | 3321 | 0.219 | 0.2801 | Yes |
| 9 | HS2ST1 | heparan sulfate 2-O-sulfotransferase 1 [Source:HGNC Symbol;Acc:HGNC:5193] | 3364 | 0.215 | 0.3095 | Yes |
| 10 | NDST4 | N-deacetylase and N-sulfotransferase 4 [Source:HGNC Symbol;Acc:HGNC:20779] | 3497 | 0.202 | 0.3300 | Yes |
| 11 | XYLT1 | xylosyltransferase 1 [Source:HGNC Symbol;Acc:HGNC:15516] | 3916 | 0.163 | 0.3223 | No |
| 12 | HS3ST3B1 | heparan sulfate-glucosamine 3-sulfotransferase 3B1 [Source:HGNC Symbol;Acc:HGNC:5198] | 4683 | 0.098 | 0.2776 | No |
| 13 | B3GAT1 | "beta-1,3-glucuronyltransferase 1 [Source:HGNC Symbol;Acc:HGNC:921]" | 4715 | 0.095 | 0.2895 | No |
| 14 | NDST3 | N-deacetylase and N-sulfotransferase 3 [Source:HGNC Symbol;Acc:HGNC:7682] | 5446 | 0.037 | 0.2385 | No |
| 15 | GLCE | glucuronic acid epimerase [Source:HGNC Symbol;Acc:HGNC:17855] | 6148 | -0.016 | 0.1865 | No |
| 16 | EXTL1 | exostosin like glycosyltransferase 1 [Source:HGNC Symbol;Acc:HGNC:3515] | 6193 | -0.020 | 0.1861 | No |
| 17 | HS3ST1 | heparan sulfate-glucosamine 3-sulfotransferase 1 [Source:HGNC Symbol;Acc:HGNC:5194] | 8162 | -0.168 | 0.0588 | No |
| 18 | HS3ST2 | heparan sulfate-glucosamine 3-sulfotransferase 2 [Source:HGNC Symbol;Acc:HGNC:5195] | 9469 | -0.271 | -0.0014 | No |
| 19 | NDST1 | N-deacetylase and N-sulfotransferase 1 [Source:HGNC Symbol;Acc:HGNC:7680] | 11241 | -0.462 | -0.0688 | No |
| 20 | HS6ST1 | heparan sulfate 6-O-sulfotransferase 1 [Source:HGNC Symbol;Acc:HGNC:5201] | 11932 | -0.579 | -0.0345 | No |
| 21 | EXTL3 | exostosin like glycosyltransferase 3 [Source:HGNC Symbol;Acc:HGNC:3518] | 12442 | -0.722 | 0.0356 | No |
Table: GSEA details [plain text format]

  

Fig 2: KEGG\_GLYCOSAMINOGLYCAN\_BIOSYNTHESIS\_HEPARAN\_SULFATE      
 Blue-Pink O' Gram in the Space of the Analyzed GeneSet

  

Fig 3: KEGG\_GLYCOSAMINOGLYCAN\_BIOSYNTHESIS\_HEPARAN\_SULFATE: Random ES distribution      
 Gene set null distribution of ES for **KEGG\_GLYCOSAMINOGLYCAN\_BIOSYNTHESIS\_HEPARAN\_SULFATE**
